# Supplementary material for: Identification of a Toxin–Antitoxin System That Contributes to Persister Formation by Reducing NAD in Pseudomonas aeruginosa
Source: Microorganisms. 2021 Apr 2;9(4):753. doi: 10.3390/microorganisms9040753 (PMC8065639; doi:10.3390/microorganisms9040753)
Supplement: Supplementary file 1 [file microorganisms-09-00753-s001.zip › Supplementary Material/Supplementary Material.docx]

**Figure S1**

A


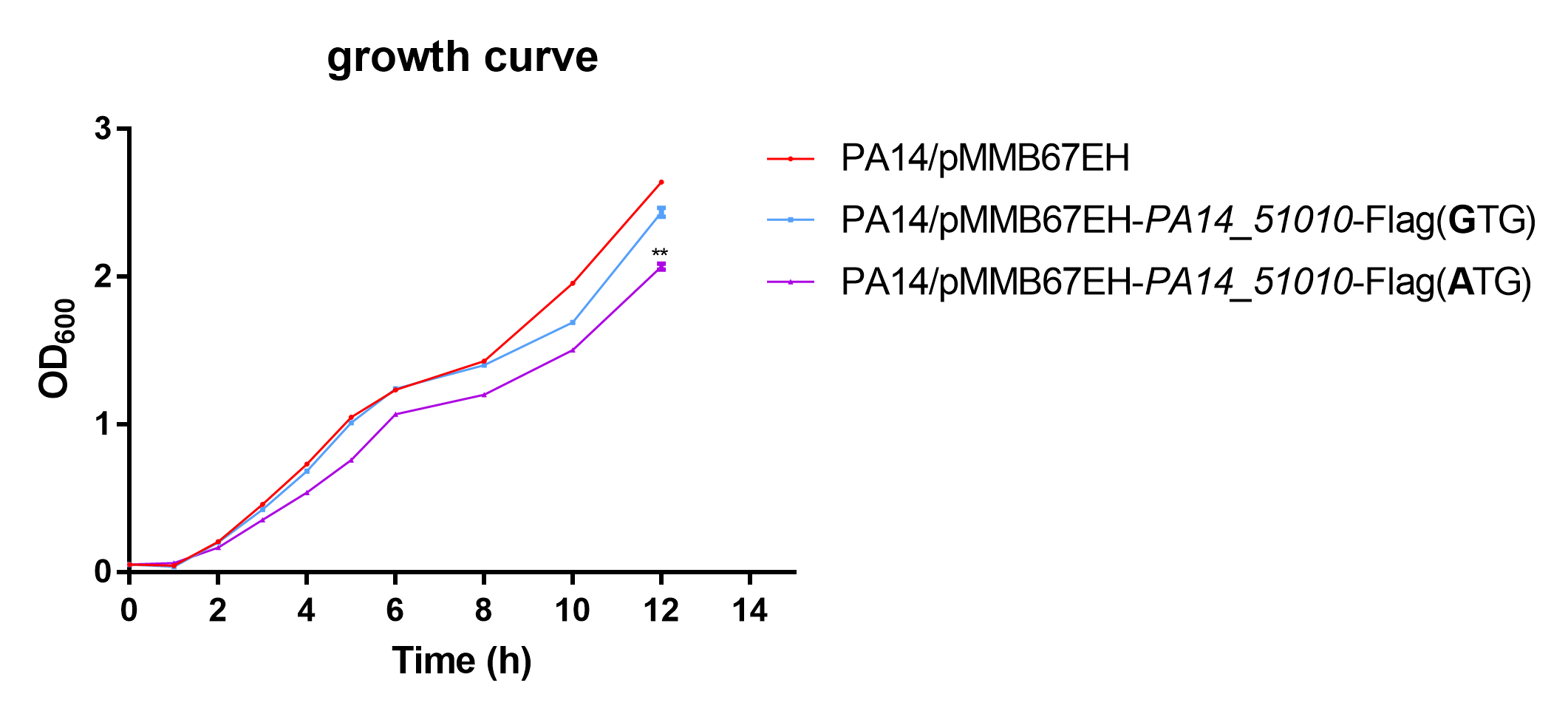


B


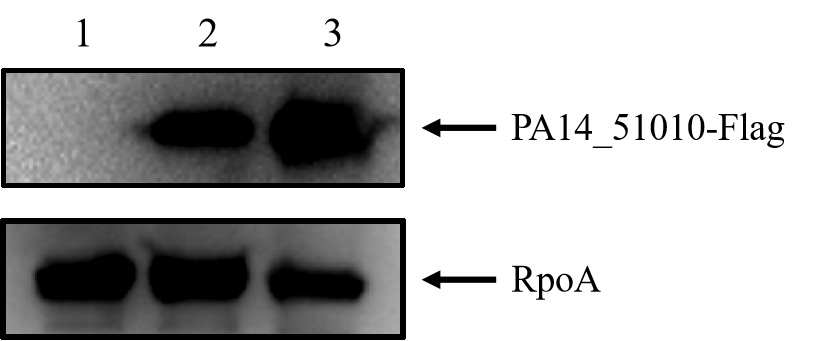


**Figure S1. Change of the start codon of the *PA14_51010* gene from the original GTG to ATG reduced the bacterial growth rate.** (A) Wild type PA14 carrying pMMB67EH, pMMB67EH-*PA14_51010*-Flag (GTG), pMMB67EH-*PA14_51010*-Flag (ATG) were cultured in LB overnight and then diluted 1:100 in fresh LB with 1 mM IPTG. At indicated time points, the OD_600_ of each of the samples was measured. **, p < 0.01 compared to the other samples by Student’s t test. (B) Protein levels of the PA14_51010-Flag in the aforementioned strains [1, PA14/pMMB67EH; 2, PA14/pMMB67EH-PA14_51010-Flag(GTG); 3, PA14/pMMB67EH-PA14_51010-Flag(ATG)]. The bacteria were cultured in LB with 1 mM IPTG to an OD_600_ of 1.0. The Flag-tagged PA14_51010 were detected by western blot with an anti-Flag antibody (Sigma) and an anti-RpoA antibody (BioLegend).
