# Supplementary figures and images for: Identification of a Toxin–Antitoxin System That Contributes to Persister Formation by Reducing NAD in Pseudomonas aeruginosa
Source: Microorganisms. 2021 Apr 2;9(4):753. doi: 10.3390/microorganisms9040753 (PMC8065639; doi:10.3390/microorganisms9040753)

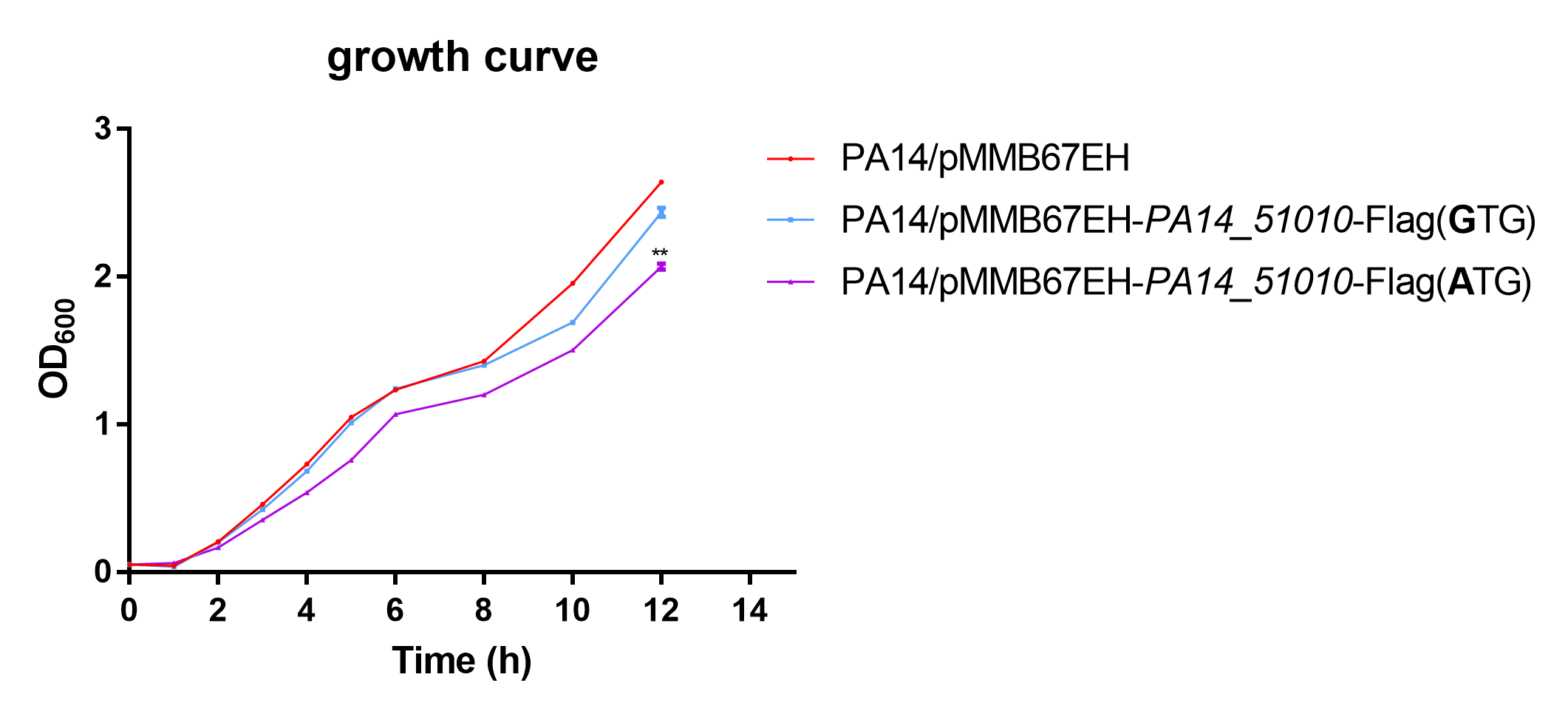

Supplement: Supplementary file 1 [file microorganisms-09-00753-s001.zip › Supplementary Material/Figures in supplementary material/Figure S1 A.tif]

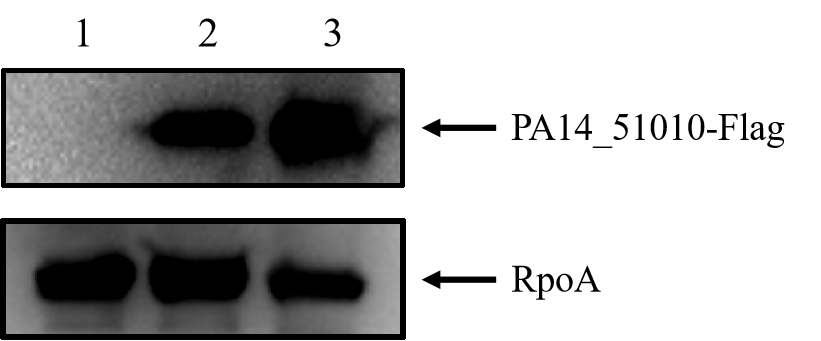

Supplement: Supplementary file 1 [file microorganisms-09-00753-s001.zip › Supplementary Material/Figures in supplementary material/Figure S1 B.tif]
